# Novel *BMP2K::PDGFRA* fusion in an unusual myeloid/lymphoid neoplasm with eosinophilia

**DOI:** 10.1007/s12308-025-00661-7

**Published:** 2025-10-24

**Authors:** Ravi Tej Bommu, Laila O. Mnayor, Mehrnoosh Tashakori, Sophia Yohe

**Affiliations:** 1 Department of Pathology, University of Iowa Health Care/Carver College of Medicine, Iowa City, IA USA; 2 Molecular Pathology and Cytogenomics, Hartford Healthcare, Hartford Hospital, Hartford, CT USA; 3 Department of Laboratory Medicine and Pathology, University of Minnesota, Minneapolis, MN USA

**Keywords:** Myeloid and lymphoid neoplasms, *PDGFRA* fusion, Novel partner, Testing methodology, *BPM2K*

## Abstract

**Background:**

Myeloid and/or lymphoid neoplasms with eosinophilia *PDGFRA* gene fusions usually occur with *FIP1L1* as the partner gene; however, novel partners have been described. These novel partners are sometimes responsive to tyrosine kinase inhibitor therapy.

**Purpose:**

We describe here a patient with a *PDGFRA* fusion with a previously undescribed partner gene, *BMP2K*.

**Methods:**

RNA sequencing with the Archer Fusion Plex Pan Solid Tumor Panel (IDT) was used to detect fusions. DNA sequencing using a custom IDT panel was also performed.

**Results:**

An in-frame *BMP2K::PDGFRA *fusion was detected in a patient who had an ill-defined myeloid neoplasm with eosinophilia. The myeloid neoplasm had a prominent mast cell component and myeloid blast component in the lymph node, while the bone marrow showed hypercellularity, eosinophilia, and myelofibrosis. DNA NGS revealed a pathogenic TP53 mutation but was negative for mutations in other genes including KIT, JAK2, CALR, and MPL.

**Conclusions:**

Given that *PDGFRA* fusions with novel fusion partners may respond to tyrosine kinase inhibitor therapy, partner agnostic testing methods should be considered either up front or as reflex testing in patients with myeloid and/or lymphoid neoplasms with blood, bone marrow, or tissue eosinophilia.

## Introduction

Platelet derived growth factor receptor alpha (*PDGFRA*) rearrangements have been described in a subset of myeloid and/or lymphoid neoplasms with eosinophilia and tyrosine kinase gene fusions (MLN-TK), commonly presenting as chronic eosinophilic leukemia but rarely presenting as other myeloid and/or lymphoid neoplasms. Most cases have the *FIP1L1::PDGFRA* fusion caused by intra-chromosomal deletion of the *CHIC2* gene fusing the 5’ end of *FIP1L1* to the 3’ end of *PDGFRA* leading to constitutive activation of *PDGFRA*. These neoplasms are exquisitely sensitive to tyrosine kinase inhibitors (TKIs). Other gene partners leading to a similar effect on *PDGFRA* have rarely been described and have shown anecdotal response to tyrosine kinase inhibitors [1–4]. We describe a case of an unusual myeloid neoplasm containing a *PDGFRA* fusion with a novel partner gene *BMP2K.*

## Clinical history

**Fig. 1:**
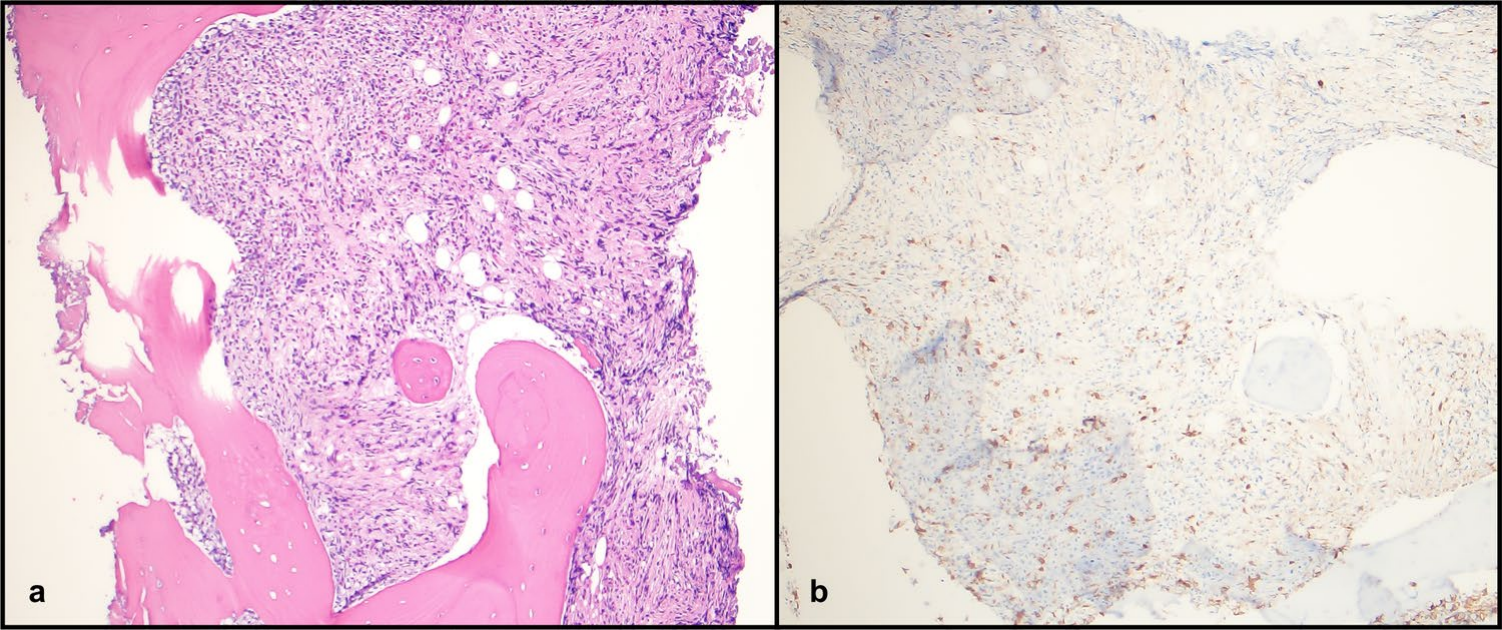
Bone marrow biopsy showing **a** hypercellular bone marrow with fibrosis, hematoxylin and eosin stain (H&E) 10 × **b** and rare scattered mast cells without clusters on CD117, 10x

A 71-year-old female with ulcerative colitis, multiple medical issues, and steroid treatment for 3 months of diffuse pain underwent bone marrow biopsy for persistent anemia. Aspirate smears were hypocellular and hemodiluted limiting testing on the aspirates. Her bone marrow was hypercellular with marked fibrosis (MF3 of 3 reticulin fibrosis) and increased eosinophils (Fig. 1). There was no increase in blasts by CD34 immunohistochemistry (IHC). CD3 and CD20 showed scattered T cells and B cells respectively. Mast cells were scattered by CD117 and mast cell tryptase. CD30 was negative. CD61 showed an increased proportion of megakaryocytes but no prominent clustering. Karyotype was unsuccessful and an NGS panel was negative for JAK2, CALR, or MPL mutations on the hemodiluted bone marrow aspirate. The working clinical diagnosis was primary myelofibrosis, she was evaluated for transplant but was deemed not to be a candidate. The patient presented 2 months later with dyspnea and persistent generalized pain. A CT scan revealed bilateral axillary lymphadenopathy, up to 4.0 cm.

**Fig. 2:**
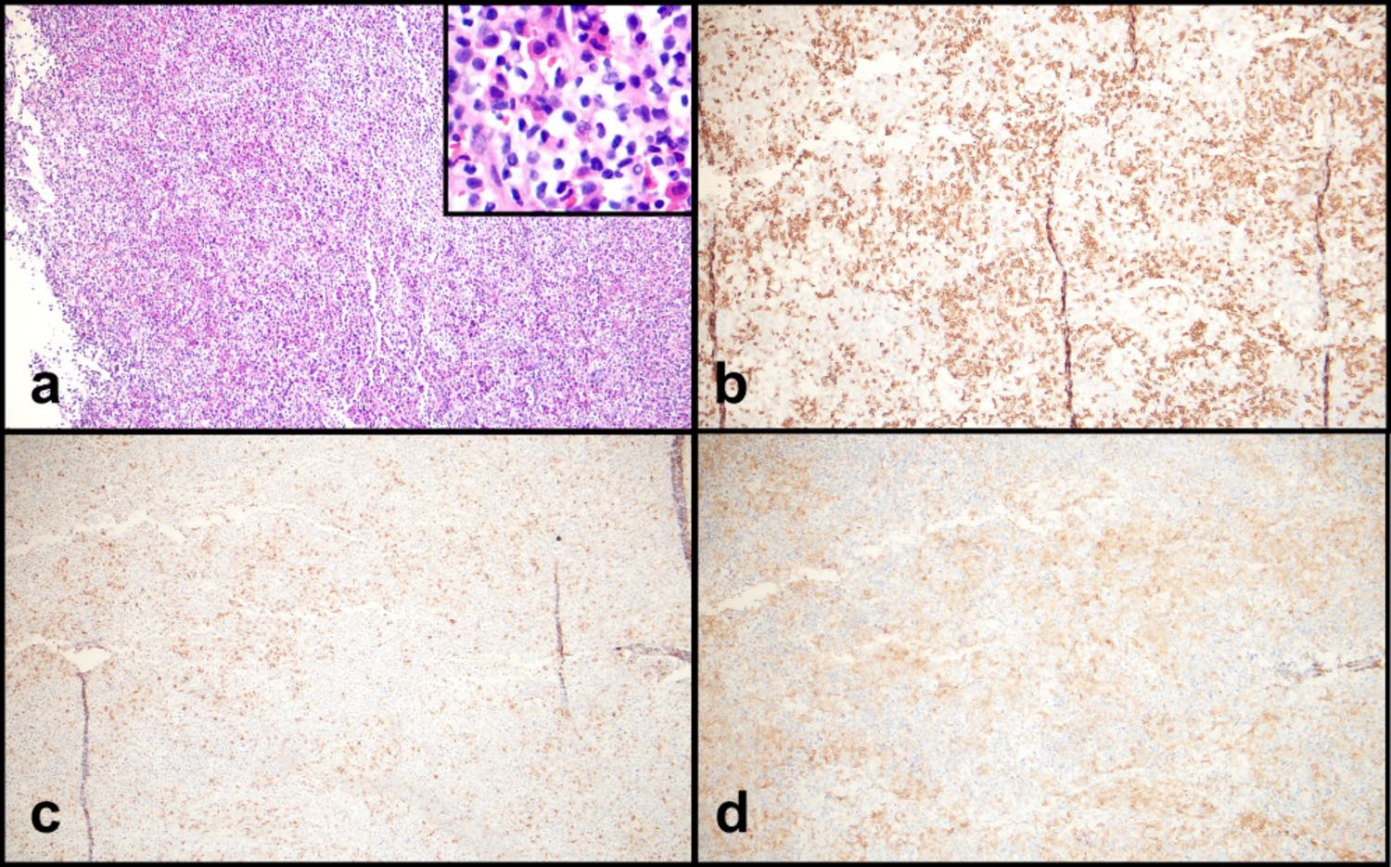
Lymph node biopsy with **a** areas of eosinophils and numerous mast cells with cleared cytoplasm, H&E 10x, inset 40x; **b** positive for CD117, 10x; **c** partial mast cell tryptase, 10x; and **d** partial CD25, 10x

**Fig. 3:**
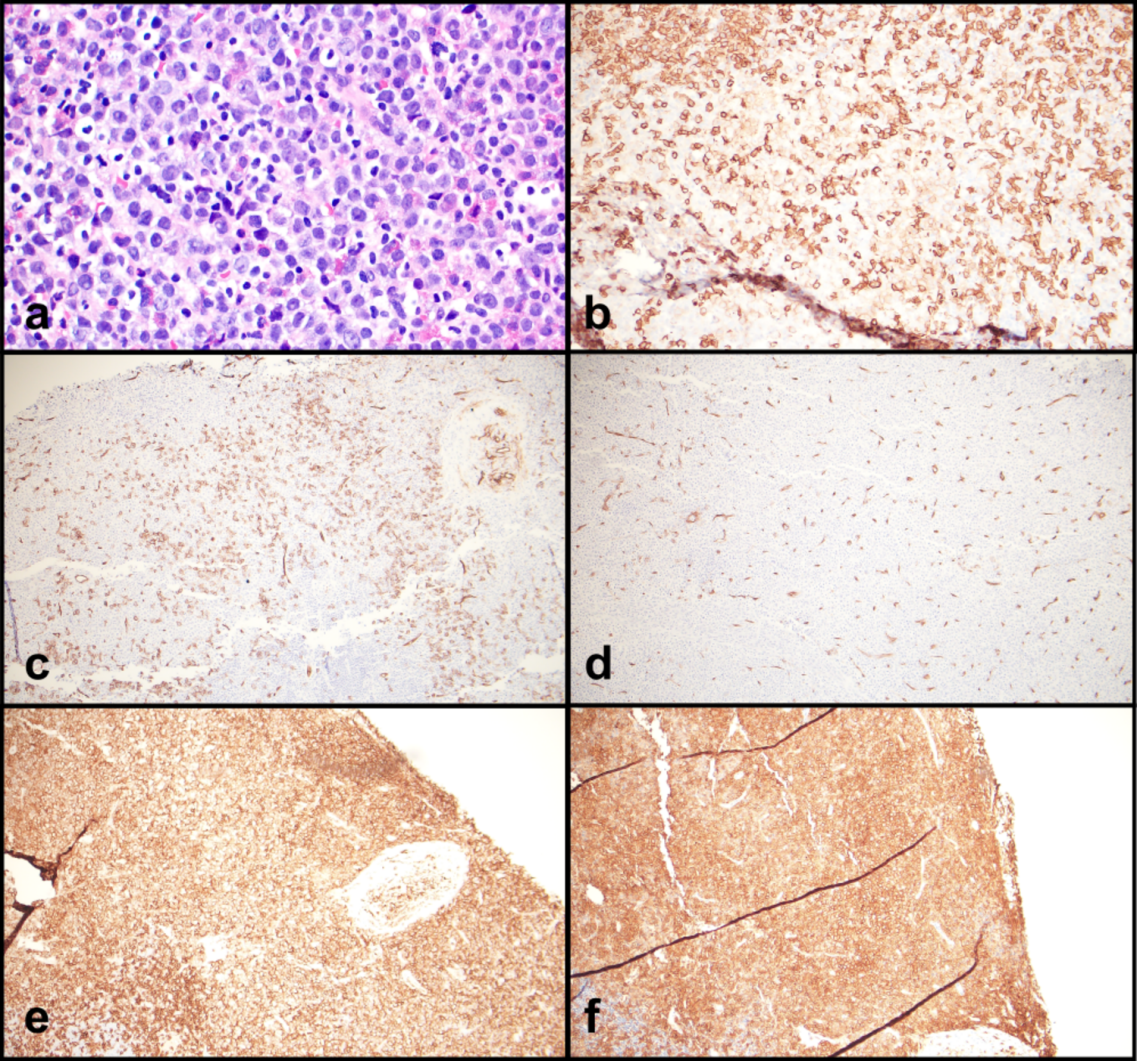
Lymph node biopsy with **a** mast cells with cleared out cytoplasm, eosinophils, and large immature cells with open chromatin, H&E 40x. **b** CD117 highlights mast cells (bright) and large immature cells (dim), 40x. **c** CD34 is present in the large immature cells, 10x. **d** CD34 in an area with fewer large cells, 10x. **e** CD4 highlighting most of the cells (mast cells and large immature cells), 10x. **f** CD33 highlighting most of the cells (mast cells and large immature cells), 10x

A right axillary lymph node biopsy was performed and demonstrated architectural effacement by a diffuse infiltrate predominantly composed of atypical mast cells, eosinophils, and scattered and clustered large immature cells (Figs. 2a and 3a). The complete blood count was significant only for anemia; there was no eosinophilia or monocytosis. A serum tryptase was slightly elevated at 15.7 ug/L.

Immunohistochemical stains showed the mast cell component to be positive for CD117, partial mast cell tryptase, and partial CD25 (Figs. 2b–d and 3b). CD34 highlighted the immature cells, predominantly in subcapsular areas (Fig. 3c and d). Nearly all the cells, including the mast cell component, were positive for CD4 and CD33 (Fig. 3e and f). MPO highlights a subset of cells with variable morphology including cells with scant cytoplasm to cells with ample cytoplasm. CD30 highlights the large cells. The infiltrate was negative for CD3, CD20, PAX5, CD56, CD61, CD68, CD123, TdT, and lysozyme. Flow cytometry identified 2.5% CD34-positive myeloid blasts and 9% abnormal mast cells with co-expression of CD117 (bright) and CD25. Molecular studies were performed.

After the lymph node biopsy, the patient was started on hydroxyurea 500 mg while ruxolitinb was considered but not started. She deteriorated further over the next month with severe debilitating pain resistant to treatment, declining functional status requiring help in the home, and dyspnea. After a novel *PDGFRA* fusion was found, the patient was started on loratidine and an appointment was made to discuss treatment options. However, with her declining status, she was transferred to palliative care within a month of the lymph node biopsy and died shortly thereafter.

## Materials and methods

### Next generation sequencing (NGS)

DNA was enriched and sequenced at the University of Minnesota, M Health Fairview Molecular Diagnostic Laboratory using a subset of DNA probes (Integrated DNA Technologies [IDT], Boulder, CO) created for the Genomics Organization for Academic Laboratories (GOAL; goalabs.org) consortium.

Extracted DNA was fragmented using the Covaris ML230 (PerkinElmer, Waltham MA). Libraries were prepared using the IDT xGen® FFPE DNA Library Prep Kit using 50 ng input. Pooled libraries are hybridized with IDT xGen™ Hybridization Capture Core Reagents probe pool of 127 genes at 100 amol/probe/µL. Enriched libraries were loaded at 10 pM concentration onto an Illumina Miseq (Illumina Inc., San Diego, CA) with 73 base pair read lengths using MiSeq Reagent Kit v3 (150-cycle). FASTQ files are processed through a custom bioinformatic pipeline. Unmapped BAM files are created from FASTQ files using fgbio software (Wellcome Trust Sanger Institute). The Picard software suite was used to collect QC and BEDtools is used to calculate read coverage. Both Vardict and Mutect2 are used to call variants at a VAF of ≥ 0.01. GenomOncology (GO) software is used for annotation and variant interpretation support.

### Archer fusion plex

The Archer Fusion Plex Pan Solid Tumor Panel (IDT) is an NGS based RNA fusion panel utilizing anchored multiplex PCR chemistry to generate target-enriched libraries for next generation sequencing. Briefly, extracted RNA was prepared using the cDNA Library Preparation Kit (Illumina) Libraries are sequenced using a Miseq (Illumina). The sequenced data is uploaded as FASTQ files to Archer Analysis software for analysis and report generation.

## Results

DNA NGS of the lymph node identified a TP53 mutation (NM_000546.5): [p.Thr125 =], c.375G > T with a variant allele frequency (VAF) of 69% and was negative for mutations in the other genes tested including KIT. This specific variant has also been shown to affect splicing and has been classified as pathogenic and likely pathogenic in ClinVar [5, 6]. 

RNA NGS via Archer fusion analysis identified an in-frame *BMP2K::PDGFRA* fusion that joined exon 15 of *BMP2K* (5’ partner) with exon 13 of *PDGFRA* (3’ partner), GRCh37 chromosomal coordinates chr4:79808438, chr4:55143555. This fusion comprised 55% of reads with 105 start sites and 799 reads.

**Fig. 4:**
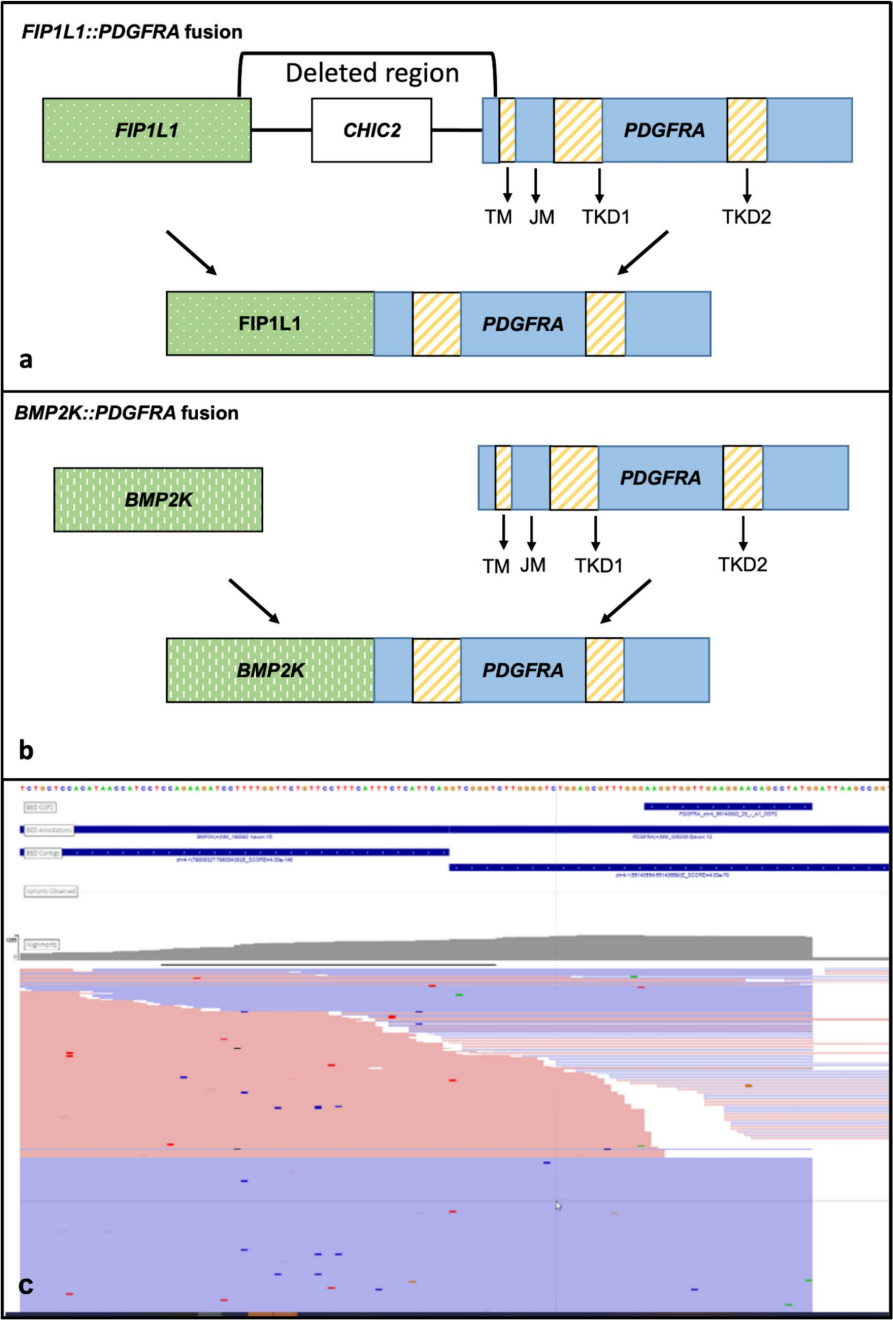
Structure of *PDGFRA* fusions: **a** genomic structure of the well-recognized *FIP1L1::PDGFRA* fusion and **b** genomic structure of the *BMP2K::PDGFRA* fusion seen in this case. **c** Visualization of RNA sequencing data showing reads spanning the fusion breakpoint

The *BMP2K::PDGFRA* fusion has the same basic structure as other *PDGFRA* fusions (Fig. 4). The *FIP1L1::PDGFRA* fusion caused by intra-chromosomal deletion of the *CHIC2* gene, fuses the 5’ end of *FIP1L1* to the 3’ end of *PDGRFA* with loss of the juxta-membrane domain and retention of the intracellular domain, including the tyrosine kinase domain, leading to constitutive activation [7]. Alternative partners have been described and have the same underlying genetic structure with retention of the 3’ end of *PDGFRA* [2, 3, 8, 9]. There is some data that *FIP1L1* contributes to the fusion’s oncogenic effect through homodimerization, nuclear localization, and/or interactions with other proteins; however, given that other fusion partners exist, these contributions either may not be required or may be fulfilled by the alternative partners [7]. *BMP2K* is a putative serine/threonine kinase containing a nuclear localization signal which theoretically could lead to nuclear localization of the *BMP2K::PDGFRA* fusion product [10].

## Discussion

We identified a novel *BMP2K::PDGFRA* fusion in a patient with an unusual myeloid neoplasm with eosinophilia and significant related morbidity including pain, dyspnea, and general debilitation. Our patient consistently lacked peripheral eosinophilia but had bone marrow and tissue eosinophilia. Myeloid and lymphoid neoplasms with eosinophilia and tyrosine kinase fusion need to be considered in this scenario as the absence of peripheral blood eosinophilia does not exclude a diagnosis. Patients with MLN-TK with *FIP1L1:: PDGFRA* are predominantly male (7:1) while our patient is female. It is not clear if there is the same distribution with PDGFRA fusions with novel partners. *BCR::PDGFRA* appears to maintain the male predominance (11 of 11 cases) [11]. In the 9 cases (including ours) with non-*FIP1L1*, non-*BCR* fusion partners 5 are male and 4 are female [2, 3, 8, 12, 13].

Although *FIP1L1* is the most recognized partner of *PDGFRA* in MLN-TK and usually presents with prominent eosinophilia, other *PDGFRA* fusion partners have been identified. These fusion partners include *BCR, CDK5RAP2, STRN, ETV6, KIF5B*, and *FOXP1* and have been described as presenting as chronic eosinophilic leukemia, chronic myeloid leukemia (CML) – like, or as atypical CML [1–4, 7–9, 11–13]. The occurrence of different *PDGFRA* fusion partners has relevance for diagnostic testing, as PCR and some FISH approaches to detecting *PDGFRA* rearrangements are specific to the *FIP1L1::PDGFRA* fusion with intra-chromosomal deletion *CHIC2* and may not detect alternative fusion partners. Several test methodologies (such as anchored multiplex PCR following by sequencing, quantitative reverse transcriptase PCR, transcriptome sequencing, FISH with break apart probes, and optical genome mapping) are available that are partner agnostic and can identify novel fusion partners [3]. Increasing use of these methods will lead to increased identification of novel partners and questions as to their biologic relevance.

An early study of *FIP1L1::PDGFRA* suggest that the critical portion of the fusion is the disruption of the juxtamembrane domain in of *PDGFRA* (exon 12, base pairs 13–106) with maintenance of the downstream 3’ tyrosine kinase domain [14]. However, a more recent study suggests that the presence of the c’terminal portion of *PDGFRA* from exon 13 is the important portion and is capable of homodimerization without a fusion partner disputing the importance of exon 12 or the *FIP1L1* fusion partner [7]. Alternative fusions partners with *PDGFRA* have been described to have the same predicted effect on *PDGFRA* with disruption or loss of the juxtamembrane domain and retained tyrosine kinase domain [1]. Although functional studies have not been performed, there is a reasonable chance that these other fusions would also lead to *PDGFRA* constitutive activation and identify patients who may benefit from tyrosine kinase inhibitor (TKI) therapy.

It is known that *FIP1L1::PDGFRA* fusion neoplasms are exquisitely sensitive to TKIs and, although data is sparse, several *PDGFRA* fusions with alternative partners (*BCR, CDK5RAP2, STRN, ETV6, KIF5B, FOXP1*) have anecdotal reports of response to TKIs [1, 2, 9, 11–13]. Since our patient did not survive for a trial of therapy we do not know whether *BMP2K::PDGFRA* would be sensitive to TKIs, but based on biology there is a fair chance that TKI therapy would have been effective.

MLN–TK with eosinophilia and *PDGFRA* fusions are rare neoplasms that may have partners other than *FIP1L1* and anecdotal evidence suggests *PDGFRA* fusions with alternate fusion partners also respond to TKI therapy. It may be time to re-evaluate the testing methodology used in the evaluation for MLN-TK that show tissue, blood, or bone marrow eosinophilia. It is important that testing methods detect alternate fusion partners so that targeted TKI therapy may be considered. Whether a tiered approach is used or whether partner agnostic methods should be used up front is an open question at this time.

## Data Availability

No datasets were generated or analysed during the current study.
